# Using Machine Learning to Characterize Atrial Fibrotic Substrate From Intracardiac Signals With a Hybrid *in silico* and *in vivo* Dataset

**DOI:** 10.3389/fphys.2021.699291

**Published:** 2021-07-05

**Authors:** Jorge Sánchez, Giorgio Luongo, Mark Nothstein, Laura A. Unger, Javier Saiz, Beatriz Trenor, Armin Luik, Olaf Dössel, Axel Loewe

**Affiliations:** ^1^Institute of Biomedical Engineering, Karlsruhe Institute for Technology, Karlsruhe, Germany; ^2^Centro de Investigación e Innovación en Bioingeniería (Ci2B), Universitàt Politècnica de València, Valencia, Spain; ^3^Medizinische Klinik IV, Städtisches Klinikum Karlsruhe, Karlsruhe, Germany

**Keywords:** atrial fibrillation, fibrosis, machine learning, bidomain, transmurality, density, cardiac modeling

## Abstract

In patients with atrial fibrillation, intracardiac electrogram signal amplitude is known to decrease with increased structural tissue remodeling, referred to as fibrosis. In addition to the isolation of the pulmonary veins, fibrotic sites are considered a suitable target for catheter ablation. However, it remains an open challenge to find fibrotic areas and to differentiate their density and transmurality. This study aims to identify the volume fraction and transmurality of fibrosis in the atrial substrate. Simulated cardiac electrograms, combined with a generalized model of clinical noise, reproduce clinically measured signals. Our hybrid dataset approach combines *in silico* and clinical electrograms to train a decision tree classifier to characterize the fibrotic atrial substrate. This approach captures different *in vivo* dynamics of the electrical propagation reflected on healthy electrogram morphology and synergistically combines it with synthetic fibrotic electrograms from *in silico* experiments. The machine learning algorithm was tested on five patients and compared against clinical voltage maps as a proof of concept, distinguishing non-fibrotic from fibrotic tissue and characterizing the patient's fibrotic tissue in terms of density and transmurality. The proposed approach can be used to overcome a single voltage cut-off value to identify fibrotic tissue and guide ablation targeting fibrotic areas.

## 1. Introduction

Atrial fibrillation (AF) is the most common cardiac arrhythmia and is characterized by an irregular heart rhythm, which is upheld by structurally altered fibrotic tissue (Platonov, 2017). Fibrosis modifies the cardiac substrate and creates a heterogeneous medium for electric propagation. Specifically, the deposition of excessive collagen fibers in the extracellular matrix affects intercellular connections, increases conduction anisotropy, and leads to slowed conduction. As such, fibrosis facilitates functional and structural conduction block, promotes reentry, and provides anchors for reentrant activity. In this way, fibrotic remodeling of the cardiac substrate favors initiation and maintenance of cardiac arrhythmia (Hinderer and Schenke-layland, 2019).

Catheter ablation is a first line therapy for patients with persistent AF (Hindricks et al., 2020). Substrate ablation strategies guided by a voltage map derived from the amplitude of intracardiac electrograms define areas based on a cut-off value (frequently <0.5 mV during sinus rhythm) as pathological tissue and target them for ablation (Malcolme-Lawes et al., 2013; Kawaji et al., 2019; Nairn et al., 2020). Several clinical studies have shown a correlation of fibrosis identified through late gadolinium enhancement magnetic resonance imaging (LGE-MRI) with reduced local signal amplitude (“voltage”) in atrial electrograms (Jadidi et al., 2013; Caixal et al., 2020). Using low voltage areas as targets for ablation has not yet shown an optimal and consistent reduction in the rate of recurrent atrial fibrillation (Verma et al., 2015; Jadidi et al., 2016; Schade et al., 2020). In addition, the interpretation of the electrograms measured at the endocardial surface of the tissue is still poorly understood, and there is no consensus about the voltage cut-off value to define arrhythmogenic substrate (Tzeis et al., 2019; Nairn et al., 2020).

In recent years, computational modeling has provided detailed insight into the mechanistic role of fibrotic tissue characteristics in the initiation and maintenance of arrhythmias (McDowell et al., 2013; Roney et al., 2016; Gokhale et al., 2017). *In silico* experiments showed that the morphology of the electrograms is related to tissue heterogeneities (Keller et al., 2014; Gokhale et al., 2017) and help to improve ablation strategies for treating AF (Lin et al., 2016; Jadidi et al., 2020). Controlled simulation environments provide the ideal setup to analyze how the fibrosis characteristics volume fraction and transmurality affect intracardiac signals and can be leveraged to pinpoint arrhythmogenic tissue.

With the increasing amount of data available, the use of machine learning for the interpretation of cardiac signals is steadily increasing. Machine learning has been extensively used in electrocardiogram analysis due to its potential to analyze big datasets and uncover mechanistic information about cardiac electrical function (Cabrera-Lozoya et al., 2017; Hannun et al., 2019; Lown et al., 2020; Luongo et al., 2020). While several studies aimed at quantifying AF mechanisms and automatically localize reentrant drivers using *in silico* or clinical electrograms (Schilling et al., 2015; McGillivray et al., 2018; Lozoya et al., 2019), less attention has been paid to the information that intracardiac electrograms can provide about the cardiac substrate based on the signal morphology due to the effect of fibrosis. Campos et al. (2013) classified different types of fibrosis based on electrogram features using *in silico* experiments. However, quantification of fibrotic volume fraction and transmurality in the atrial substrate has not been reported yet to the best of our knowledge. Additionally, data-driven approaches can help to overcome the use of a single voltage cut-off value to characterize the cardiac substrate and distinguish between non-fibrotic and fibrotic tissue based on a more comprehensive, holistic set of criteria.

We aim to quantify the volume fraction and transmurality of fibrosis present in the cardiac tissue by machine learning on features extracted from intracardiac electrograms. In the current state, clinical electrograms do not provide information to directly characterize the fibrotic substrate. Therefore, we created highly-detailed multi-scale biophysical simulations that capture the electrogram signature of fibrotic tissue. Additionally, clinical electrograms from high voltage areas and low complexity captured the variability of healthy tissue. Combined with the simulated electrograms, they formed the basis of a hybrid dataset to train a machine learning algorithm based on features extracted from intracardiac signals to characterize the atrial substrate.

## 2. Materials and Methods

**Figure 1:**
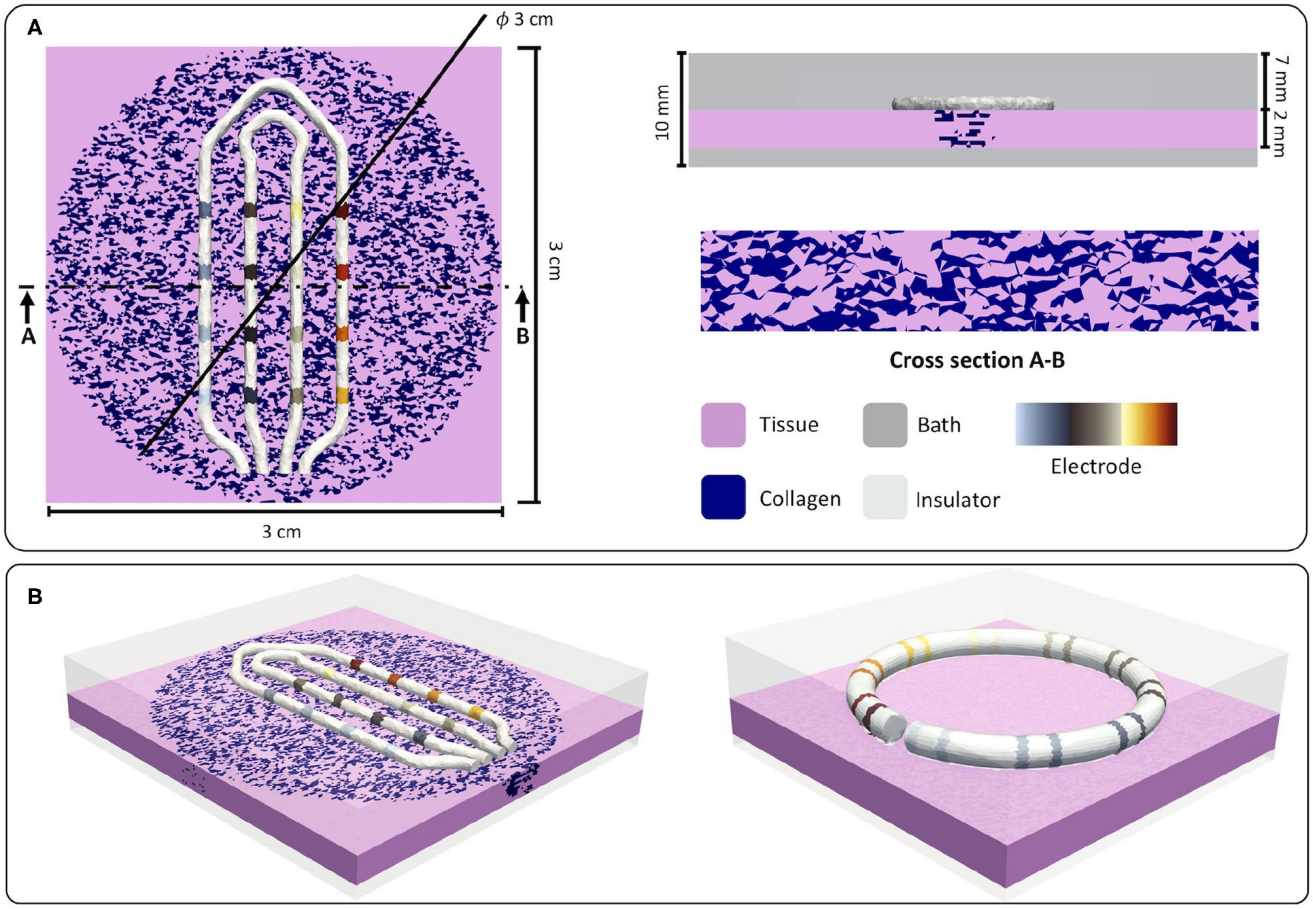
**(A)** Dimensions of the *in silico* setup. Tissue dimensions, catheter position, and fibrotic dimensions are shown in the left panel. In the top right corner, bath dimensions are depicted. A cross-section cut showing the interstitial model is depicted in the lower right corner. **(B)** Isometric view of the two setups used for the *in silico* experiments. The left panel shows the setup using the HD Grid catheter (St. Jude Medical, EnSite HD Grid catheter, St. Paul, MN) and the fibrotic tissue. The right panel shows the setup using the Lasso catheter (Biosense Webster, Diamond Bar, CA, USA) on top of the tissue. Reused from Sánchez et al. (2021).

We created unstructured meshes to represent a patch of cardiac tissue surrounded by a bath (blood). On top of the tissue, we placed realistic models of two commercially available intracardiac mapping catheters, as depicted in Figure 1. Fibrosis was modeled with different densities and transmurality within a circular area in the center of the patch.

### 2.1. Tissue Setup

Tissue patch dimensions were 30 × 30 × 2 mm with an average element length of 200 μm, as shown in Figure 1A. To address the variability that ionic models could introduce in the calculation of electrograms, we used two different ionic models to simulate the electrophysiology of the human atrial tissue to generate the hybrid dataset. Human atrial cellular electrophysiology was characterized by the mathematical models proposed by Courtemanche et al. (1998) and Koivumaki et al. (2009). To reproduce the electrical remodeling in cells due to persistent atrial fibrillation, the Courtemanche et al. (1998) model was modified as suggested (Loewe et al., 2014), whereas the Koivumaki et al. (2009) model was modified according to Skibsbye et al. (2016). Cardiac bidomain conductivity ratio between the intracellular and the extracellular medium was adjusted in a tissue strand in two scenarios to achieve plane wave conduction velocities of 30 and 40 cm/s (McDowell et al., 2013). To consider different directions of electrical propagation, the tissue was stimulated from three sides: left border, bottom border, and top right corner.

### 2.2. Fibrosis Modeling

Several studies have shown the importance of the texture of the fibrotic tissue for excitation propagation in the cardiac substrate (Jakes et al., 2019; Dokuchaev et al., 2020; Nezlobinsky et al., 2020). Our proposed model aims at reproducing the deposited collagen fibers observed in tissue samples with interstitial fibrosis (Hansen et al., 2017). Fibrotic infiltrations were grown from the endocardial side to the epicardium with three different degrees of transmurality: 0.5, 1, and 2 mm (i.e., fully transmural). Fibers of collagen were placed following uniform distributions by labeling mesh elements as collagen. Collagen was modeled as low conductive extracellular medium with a conductivity of 1 × 10^−6^ S/m (Lima et al., 2006; Keller et al., 2014) and an average length of 600 ± 200 μm (Jacquemet and Henriquez, 2009; Eduardo et al., 2016). Conductivity of myocytes within the circular fibrotic region was reduced by 53% in the longitudinal direction and increased 2.5-fold in the transverse direction to simulate the effect of gap junction reduction observed during persistent AF (McDowell et al., 2013). Ten different random realizations were considered per density and transmurality.

### 2.3. Electrogram Signals

To represent the effect of the catheter geometry on the electrogram, we incorporated two realistic geometries of commercially available catheters as depicted in Figure 1B. The left panel in Figure 1B shows the geometry of an HD Grid catheter (St. Jude Medical, EnSite HD Grid catheter, St. Paul, MN), and the right panel shows the geometry of a Lasso catheter (Biosense Webster, Diamond Bar, CA, USA) with an interelectrode distance of 2 mm between electrodes of one pair and 6 mm between pairs. Electrodes were modeled as a highly conductive material (1 × 10^12^ S/m) while insulator materials were modeled as low conductance (1 × 10^−6^ S/m).

**Figure 2:**
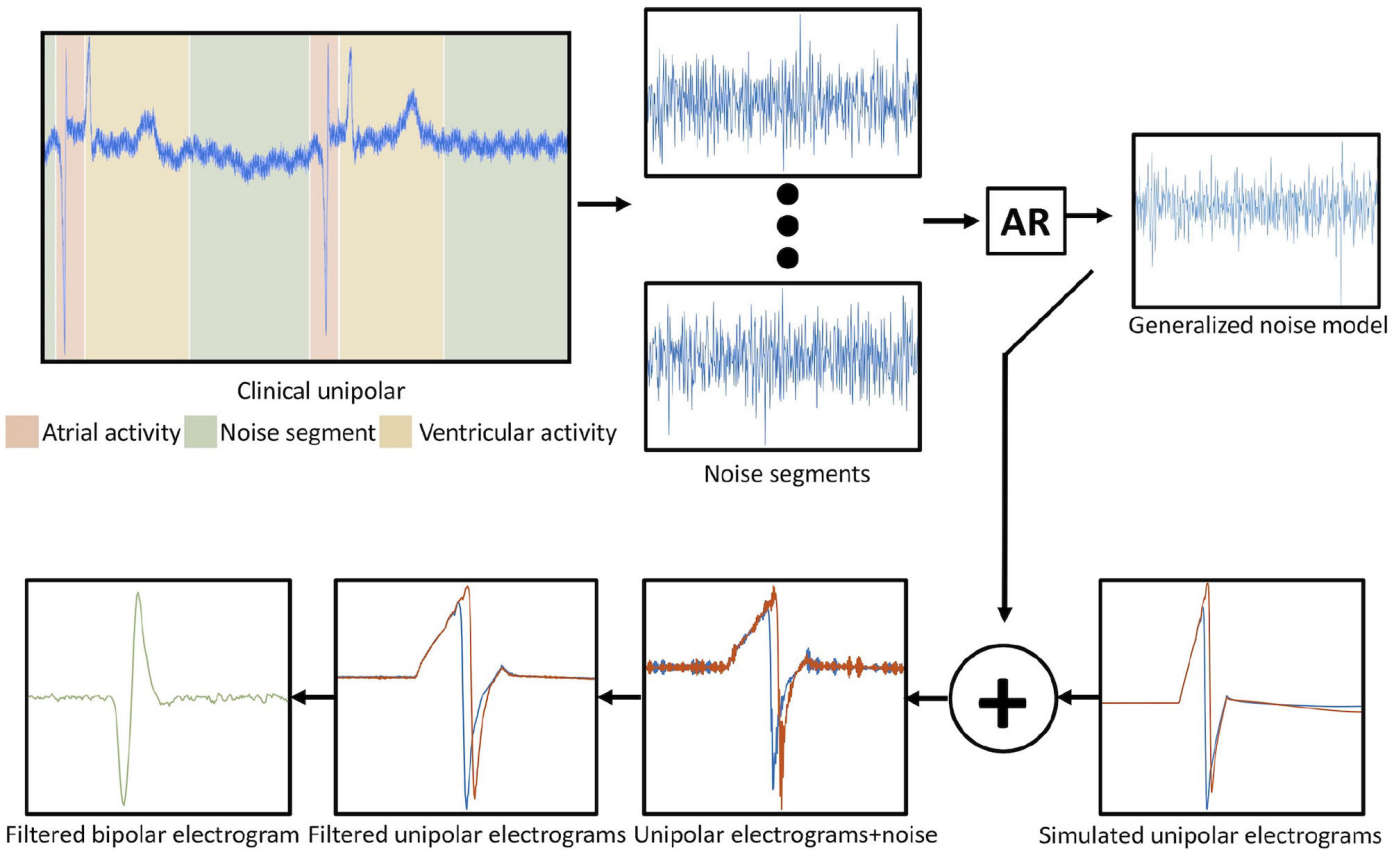
Workflow to generate the noise model and the addition to the simulated signals. In the top left corner, the different segments of the activity from a clinical unipolar electrogram are depicted. Autoregression was applied to the noise segments. The noise model was used to estimate the simulated unipolar electrogram with noise. Afterward, the unipolar electrograms (red and blue trace) were filtered, and the bipolar electrogram was calculated by subtracting the unipolar electrograms. Reused from Sánchez et al. (2021).

Unipolar electrograms, sampled at 2 kHz, were extracted from the bidomain simulations for every electrode of the catheter. Additionally, a generalized model of noise extracted from clinical signals from the four patients in the training set was created using an autoregressive approach and added to the simulated unipolar signals as depicted in Figure 2. First, ventricular far-fields were blanked from the unipolar clinical signals as well as atrial activity, thus keeping only the noise segments. The noise model was created from thirteen extracted noise segments from unipolar clinical signals. Each segment was fitted using an autoregressive model:

(1) $$\begin{array}{r} X_{t}=\overset{p}{\underset{i=1}{\sum }}\phi_{i}X_{t-i}+\epsilon_{t}^{*}\;, \end{array}$$

where *X*_*t*_ is the time series and $\epsilon_{t}^{*}$ is white noise. The model order p was determined based on the Bayesian information criterion. The smallest Akaike information criterion value determined the global order, and the model coefficients ϕ_*i*_ were averaged to obtain a global model representing the clinical noise of intracardiac signals. The generalized model was added to the simulated unipolar signals as depicted in Figure 2.

After adding noise, both unipolar and bipolar signals were filtered using a Butterworth second order band-pass filter implemented in Matlab. Unipolar synthetic signals were filtered using a band-pass between 0.05 and 900 Hz. Afterward, bipolar electrograms were calculated by subtracting the signals from the corresponding pairs of electrodes and filtered by a clinically used band-pass filter between 30 and 300 Hz (Deno et al., 2017; Unger et al., 2019).

### 2.4. Numerical Simulations

Biophysical simulations were run with openCARP (Vigmond et al., 2003; Sánchez et al., 2020) using a full bidomain model described in Equations (1) to (6), which provides the most physiologically-realistic representation of cardiac bioelectric activity. The bidomain model accounts for bath-loading effects by representing a surrounding extracellular bath and the physical properties of the electrode as an equipotential material.

(2) $$\begin{array}{r} \nabla \cdot \left(\sigma_{i}\nabla \phi_{i}\right))=\beta I_{m} \end{array}$$

(3) $$\begin{array}{r} \nabla \cdot \left(\sigma_{e}\nabla \phi_{e}\right))=-\beta I_{m}-I_{e,s} \end{array}$$

(4) $$\begin{array}{r} I_{m}=C_{m}\frac{\partial V_{m}}{\partial t}+I_{ion}\left(V_{m},\nu \right)-I_{trans} \end{array}$$

(5) $$\begin{array}{r} V_{m}=\phi_{i}-\phi_{e} \end{array}$$

ϕ represents the electrical potential, the indices *i* and *e* refer to the intracellular and extracellular spaces, respectively. σ is the conductivity tensor, β is the surface to volume ratio of the myocytes, and *I*_*ion*_ the total transmembrane ionic current density from the cellular model. The latter is dependent on *V*_*m*_ and a vector ν of further state variables. *I*_*trans*_, a transmembrane current density stimulus, and *I*_*e, s*_, an extracellular current density, describe external stimuli. If a bath surrounds a tissue, the bath is treated as an extension of the extracellular space.

Adding (2) and (3) and incorporating it into (5) yields:

(6) $$\begin{array}{r} \nabla \cdot \left(\sigma_{i}+\sigma_{e}\right)\nabla \phi_{e}=-\nabla \cdot \left(\sigma_{i}\nabla V_{m}\right)-I_{e,s} \end{array}$$

(7) $$\begin{array}{r} \nabla \cdot \left(\sigma_{i}\nabla V_{m}\right)=-\nabla \cdot \left(\sigma_{i}\nabla \phi_{e}\right)+\beta I_{m} \end{array}$$

The *in silico* model was verified and validated by applying the criteria suggested in the ASME VV-40-2018 standard of the American Standard Association of Mechanical Engineers (ASME V&V 40, 2018). The risk-informed matrix assesses the model influence in characterizing the atrial substrate using intracardiac signals. The software solution was verified as described by Niederer et al. (2011). The simulated signals were compared with clinical signals. Additionally, we considered uncertainty by simulating different propagation scenarios, including realistic geometries of two commercially available catheters, and implementing 10 different realizations per fibrosis density and transmurality for random, uniformly distributed collagen. Single cells were stimulated at a basic cycle length of 600 ms for 100 cycles. The state of the cell model at the last time step was used as the initial state for the cells in tissue level simulations. Tissue simulations were stimulated with five pulses at a basic cycle length of 600 ms. Electrograms were evaluated for the last cycle. We performed a total of 1,444 full-bidomain simulations to build the dataset of synthetic signals with a length of 2.5 s. The meshes used in this study had an average of 2 million elements and 345,000 points. The total number of electrograms included in the hybrid dataset was 2,338, of which 1,198 were clinical signals and 1,140 were simulated signals.

### 2.5. Classification Algorithm

We implemented decision tree classifiers trained to predict binary or multiclass responses for tissue characteristics in three settings: (i) fibrotic vs. non-fibrotic tissue, (ii) several degrees of fibrosis density (10, 20, 40, and 60%), and (iii) subendocardial, partially transmural, and fully transmural fibrosis.

**Figure 3:**
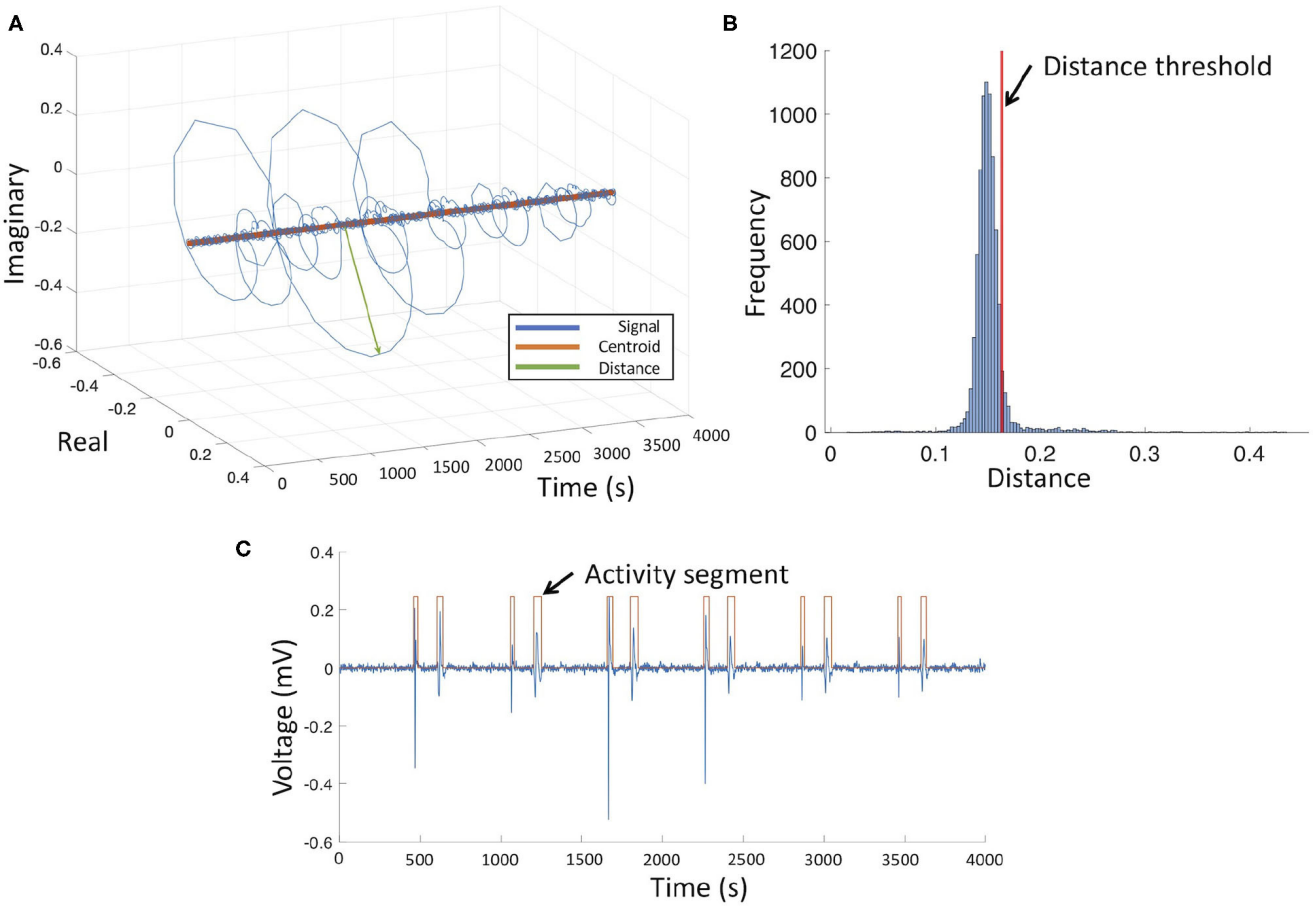
Electrogram activity detection in the Hilbert space. **(A)** Electrogram signal **(C)** in the Hilbert Space with centroid (orange trace), green arrow depicts the distance measured from the centroid to the signal. **(B)** Frequency distribution of centroid to signal distance, red line represents mean value plus one standard deviation. **(C)** Bipolar electrogram (blue trace) and activity segments (orange trace).

As input features for the decision tree, we complemented the peak-to-peak amplitude of the electrogram signal by a set of complexity measures derived from the electrograms as a signature of the fibrotic substrate and its microstructure (Figure 1A). Complexity features were extracted from the activity segments detected in the intracardiac signal to train the classifier. For each signal, segments of atrial activity were calculated by tracking closed loops in Hilbert space. The distribution of the radius of every single loop was calculated and the mean value plus one standard deviation was chosen to distinguish between cardiac activity and noise (Figure 3). The peak-to-peak amplitude was calculated for each active segment. Signal complexity was quantified for each segment of atrial activity using different entropy measures: sample entropy (Richman and Moorman, 2000), Shannon entropy (Shannon, 1948), spectral entropy (Vanluchene et al., 2004), and Kolmogorov complexity (Kolmogorov, 1968). Additionally, the fractal dimension coefficient was calculated for the whole 2.5 s signal segment (Muller et al., 1992).

The hybrid dataset was created by combining simulated electrograms and clinical electrograms and used to train the classifier. Specifically, the class of non-fibrotic synthetic signals was extended by clinical signals annotated as high voltage (peak-to-peak amplitude >0.5 mV) by a medical expert extracted from four patients. Moreover, the other five patients were used to test the classifier as a proof of concept. *In silico*, non-fibrotic tissue was simulated using two different conduction velocities (30 and 40 cm/s) to capture the effect of conduction velocity variability on peak-to-peak amplitude and active segment duration. We split the dataset into training, validation, and test sets as a 70/15/15% random split. All classes were guaranteed to be in all subsets. The validation set was used by the greedy technique to optimally tune the classifier. Furthermore, validation set accuracy was used to check that the algorithm is not overfitting when comparing against the test set accuracy. One hundred different realizations were run using hold-out cross-validation to obtain the mean accuracy of each one of the three decision tree classifiers.

The feature set considered for each classifier was selected using a greedy forward selection method (Edmonds, 1971). This iterative method starts with an empty feature set and adds the feature, which leads to the highest accuracy increase of the classifier in each iteration. The algorithm stops when performance based on the validation set cannot be further improved. Candidate features with a correlation coefficient >0.6 with any of the features already included in the set were removed. The correlation threshold was chosen as a compromise between avoiding redundant information and covering all physiologically relevant phenomena. The performance of the classification algorithm was evaluated using confusion matrices and accuracy. The classifiers were implemented in Matlab (The Mathworks, Natick, MA, USA).

### 2.6. Statistical Analysis

Data are expressed as mean ± standard error. Differences between group means were examined using two-tailed, paired Student's *t*-test and were considered significant when *p* < 0.05. The Sørensen-Dice index was used to measure the similarity between clinical low/high voltage map and the non-fibrotic vs. fibrotic map.

### 2.7. Clinical Data

This study includes nine patients recruited at Städtisches Klinikum Karlsruhe with the diagnosis of persistent AF. Patients were split into two groups; four patients were used to extract the clinical noise from the unipolar signals and train the machine learning algorithm. The other five patients were used as a proof of concept to test our approach to characterize the atrial tissue from clinical electrograms. Electroanatomical maps were acquired during sinus rhythm using the CARTO3 mapping system (Biosense Webster, Diamond Bar, CA, USA) with the Lasso catheter (Biosense Webster). The study was approved by the Institutional Review Board of Freiburg University in accordance with the Helsinki declaration. All patients gave written informed consent.

## 3. Results

### 3.1. Electrogram Features

Following the ASME V&V 40 standard (ASME V&V 40, 2018), we created highly-detailed *in silico* experiments to study the impact of structural remodeling due to AF on electrogram morphology. Bidomain simulations combined with a generalized intracardiac clinical noise model faithfully reproduced clinical recordings, which, combined with *in vivo* electrograms, were used to create the hybrid dataset.

**Figure 4:**
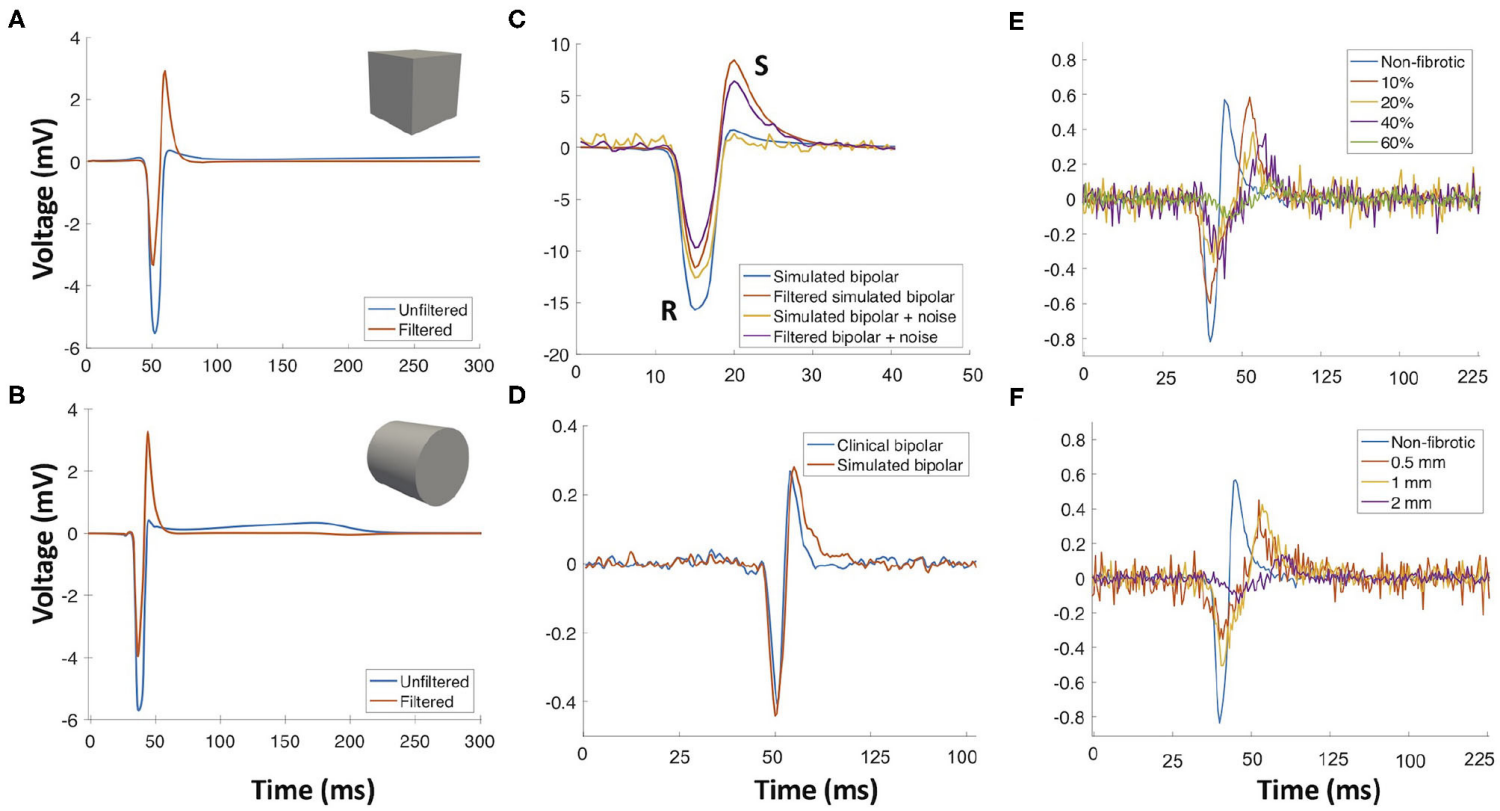
Importance of using a realistic electrode geometry and adding noise for simulated intracardiac signals. **(A)** Bipolar electrogram signal recorded with a cubic electrode (blue trace) and the corresponding filtered signal (red trace). **(B)** Signal recorded with a cylindrical electrode (blue trace) and the resulting signal after filtering (red trace). **(C)** Simulated signals recorded with a cylindrical electrode with and without noise and the resulting signals after filtering. **(D)** Comparison of a simulated signal with a clinical signal. **(E)** Electrograms recorded on the surface of the fibrotic tissue with different densities. **(F)** Effect of fibrosis growth from the endocardial surface to the epicardium (0.5, 1, 2 mm) on the electrogram (60% fibrosis density). Reused from Sánchez et al. (2021).

Modeling interstitial fibrotic texture allowed to study electrogram characteristics resulting from fibrotic tissue alterations. Fibrosis texture had a considerable impact on the electrical propagation in the tissue and on electrogram morphology (Figures 4E,F). Duration of atrial activity, which corresponds to the total activation time of the tissue underneath the electrode, calculated in Hilbert space, was increased (23.72 ± 0.05 ms) for low fibrosis density (10 and 20%) with respect to the activity duration of electrograms from non-fibrotic tissue (17.5 ± 0.04 ms). For mid-density fibrosis (40%), duration was further increased (43.80 ± 0.01 ms) and high-density fibrosis (60%) had the longest activity duration (55.31 ± 0.02 ms). Low-density fibrosis (10 and 20%) had less impact on the signal amplitude (1.08 ± 0.01 mV) compared to mid-density fibrosis (40%) which decreased the amplitude (0.83 ± 0.01 mV). High-density fibrosis (60%) had a small amplitude (0.59 ± 0.004 mV; Figure 4E). Figure 4F shows the effect of fibrosis transmurality for high density of fibrosis (60%). Subendocardial and partially transmural fibrosis (0.5 and 1 mm, respectively) had a small effect on the electrogram morphology while total transmurality (2 mm) decreased signal amplitude and prolonged its duration. The model of interstitial fibrosis yielded reduced conduction velocity reflected by an increased duration of active segments depending on the density and transmurality of fibrosis.

**Figure 5:**
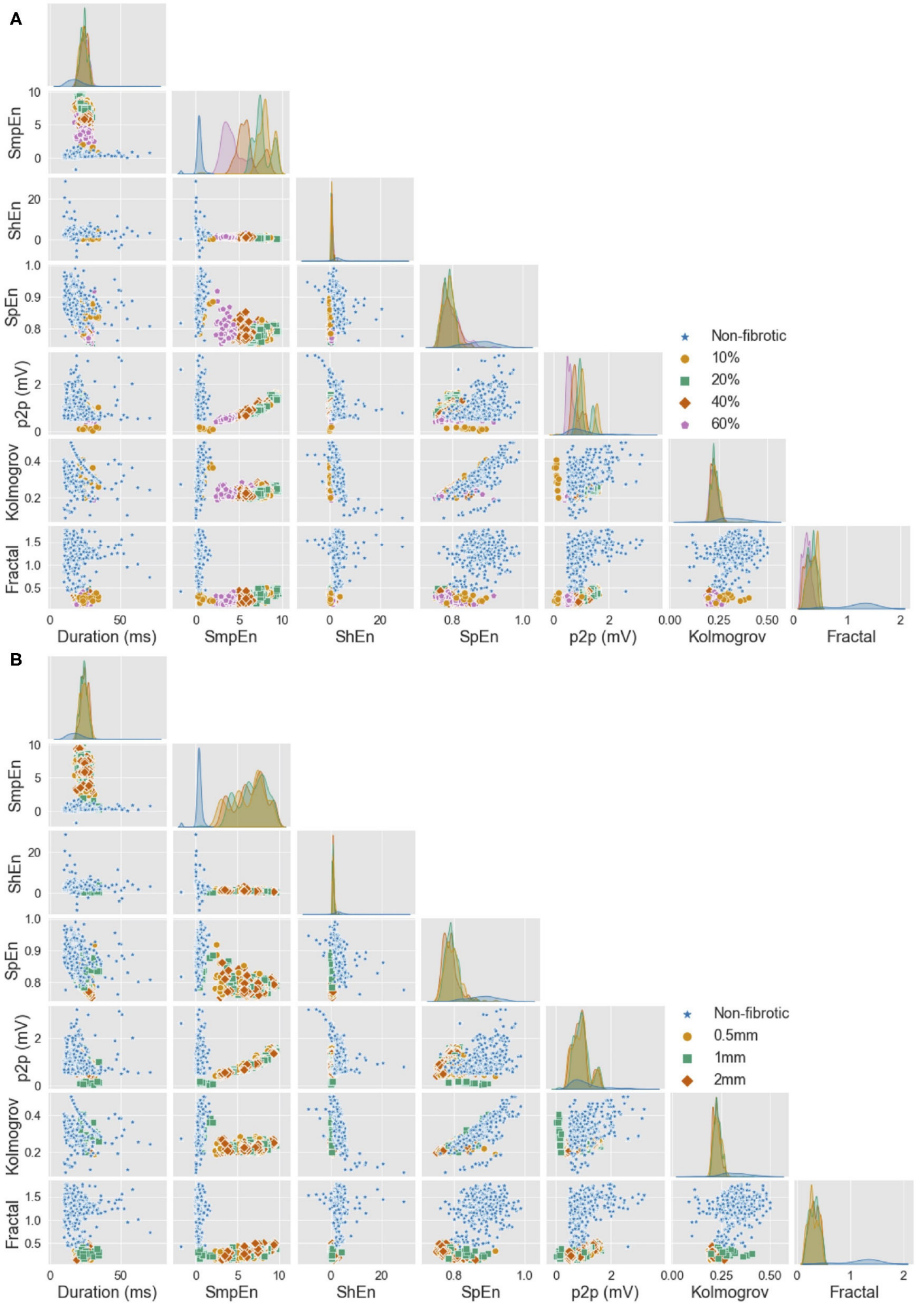
Feature distribution for all *in vivo* and *in silico* electrograms (including noise). Single feature distribution can be observed in the diagonal and the combination of two features is reflected in the scatter plots. **(A)** Features split by different densities of fibrosis. **(B)** Features split by different degrees of transmurality. Duration, duration of the active segment (ms); SmpEn, sample entropy; ShEn, shannon entropy; SpEn, spectral entropy; p2p, peak-to-peak amplitude (mV); Kolmogorov, Kolmogorov complexity; Fractal, fractal dimension. Reused from Sánchez et al. (2021).

In total, seven features to measure complexity and morphological characteristics of the signals were calculated from the bipolar electrograms (Figure 5). Features were extracted from the simulated signals with and without noise. Sample entropy and spectral entropy were robust to the addition of noise. Sample entropy value, for electrograms of non-fibrotic tissue, did not significantly change (0.18 ± 0.01 vs. 0.21 ± 0.01, *p* > 0.05). Kolmogorov complexity was less affected by noise than Shannon entropy. Shannon entropy and fractal dimensions did not perform well after the addition of noise. Shannon entropy was 0.57 ± 0.01 without noise and 3.33 ± 0.01 after adding noise to the signal (*p* < 0.05). The same behavior was observed for the fractal dimensions where the value changed from 1.15 ± 0.01 without noise to 10.2 ± 0.05 with noise (*p* > 0.05). Additionally, the duration and amplitude of the signal were considerably altered by noise.

Our *in silico* electrograms were validated against clinical electrograms recorded from areas of the atria with peak-to-peak amplitudes higher than 0.5 mV. Cross-correlation was used to align the clinical signals and simulated electrograms in time for maximal similarity. Simulated bipolar signals for non-fibrotic tissue had a mean correlation of 91.13 ± 0.05% with the clinical signals. Clinical high voltage (peak-to-peak >0.5 mV) and simulated control electrograms (no fibrosis) had a mean peak-to-peak voltage of 1.67 ± 0.05 and 2.25 ± 0.01 mV, respectively. Clinical and simulated control electrograms had a mean duration of 18.30 ± 0.56 and 17.5 ± 0.04 ms, respectively. Using realistic geometries to represent the electrodes changes the simulated electrogram morphology. Figure 4A shows a simulated bipolar electrogram simulated with cubic electrodes where the impact of filtering on the positive slope becomes visible. Figure 4B shows a simulation with a cylindrical electrode geometry mimicking the commercial catheters used in this study. The resulting electrogram is not symmetric and filtering has no marked effect on the positive slope, which is steeper than in the electrogram simulated with the cubic electrodes. Adding noise to the simulated signals decreases their amplitude and fractionates the morphology (Figure 4C). Simulated bipolar electrograms without noise have a higher amplitude of R and S peaks, which decrease with the addition of noise. Figure 4D compares a simulated signal with a clinical signal. Simulated electrogram negative and positive slopes are close to the values of the clinical signal, 0.1 and 0.25 mV/ms, respectively.

### 3.2. Classification of Tissue Characteristics

Extracted features from the bipolar electrograms are depicted in Figure 5. The main diagonal shows the distribution of the calculated features for the different groups of signals (different fibrotic densities in Figure 5A, different degrees of fibrosis transmurality in Figure 5B). Peak-to-peak amplitude is not a good feature to determine the degree of fibrosis due to the wide range of amplitudes that overlap for fibrotic vs. non-fibrotic cases. While sample entropy can distinguish between fibrotic and non-fibrotic tissue, the distribution of the values overlaps for different densities. The distinction between different fibrosis transmuralities is not possible by using only one feature since the value for all features overlap for all density or transmurality values (Figure 5B, main diagonal). Scatter plots in Figure 5 show how a combination of two features might help to characterize the fibrotic substrate. For fibrosis density, scatter plots show how combining complexity measures and commonly used features like peak-to-peak amplitude or duration of the active segment can help to differentiate non-fibrotic from fibrotic tissue.

**Figure 6:**
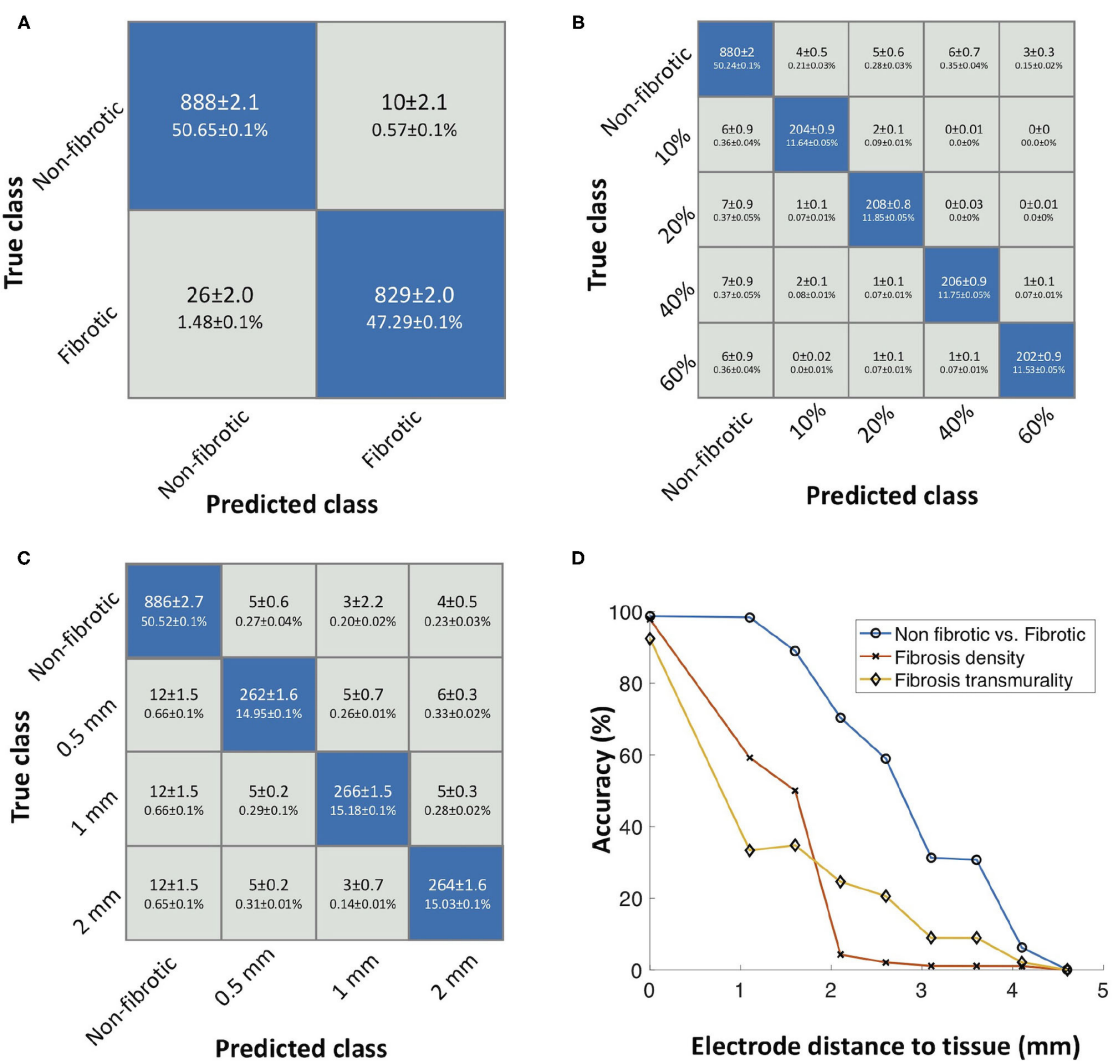
**(A)** Confusion matrix of the decision tree classifier for identifying non-fibrotic vs. fibrotic substrate. **(B)** Confusion matrix showing the performance for identifying different fibrosis densities. **(C)** Confusion matrix showing the performance for identifying transmurality of fibrosis. **(D)** Effect of increasing the electrode surface to tissue surface distance on the accuracy of the classifiers to distinguish fibrotic tissue, density, and transmurality. Reused from Sánchez et al. (2021).

A decision tree classification algorithm was trained to separate different fibrosis densities and degrees of transmurality. The combination of signal complexity features was determined by a greedy forward algorithm. The dataset was randomly divided into 70% train, 15% test, and 15% validation. The mean accuracy of the three classifiers was calculated by doing 100 different realizations. Figure 6A shows the confusion matrix of the classifier for distinguishing between non-fibrotic and fibrotic tissue. The mean accuracy for this classifier is 97.95 ± 0.03% with 98.81 ± 0.01% sensitivity and 97.16 ± 0.01% specificity. The classifier slightly overestimated the fibrotic areas. Figure 6B shows the classifier performance to identify fibrosis density (non-fibrotic, 10, 20, 40, and 60%) with a mean accuracy of 97.01 ± 0.02% and 96.33 ± 0.03% and 99.05 ± 0.01%, for sensitivity and mean specificity, respectively. The most relevant features for classification of fibrosis density, determined by the greedy forward algorithm, were sample entropy and spectral entropy (Figure 5).

To identify transmurality of fibrosis in the tissue, the classifier yielded a mean accuracy of 94.62 ± 0.01, 92.99 ± 0.02% sensitivity, and 97.86 ± 0.01% specificity. For fibrosis transmurality, misclassification occurred for some cases. Nevertheless, it is able to distinguish all four classes (non-fibrotic, 0.5, 1, and 2 mm). The most relevant features for classification of transmurality were sample entropy and peak-to-peak amplitude.

Furthermore, we investigated the effect of increasing the distance between the catheter and atrial endocardial surface and the classifiers' accuracy. The classifier's accuracy dropped with increased distance, as shown in Figure 6. The accuracy of the classifier dropped to 0% for electrode-to-tissue distances bigger than 4.1 mm, to distinguish non-fibrotic from fibrotic tissue. For identifying different densities, the accuracy dropped to 59.17% at 1.1 mm distance to tissue. Additionally, transmural accuracy drops to 33.30% with a distance to tissue of 1.1 mm.

**Figure 7:**
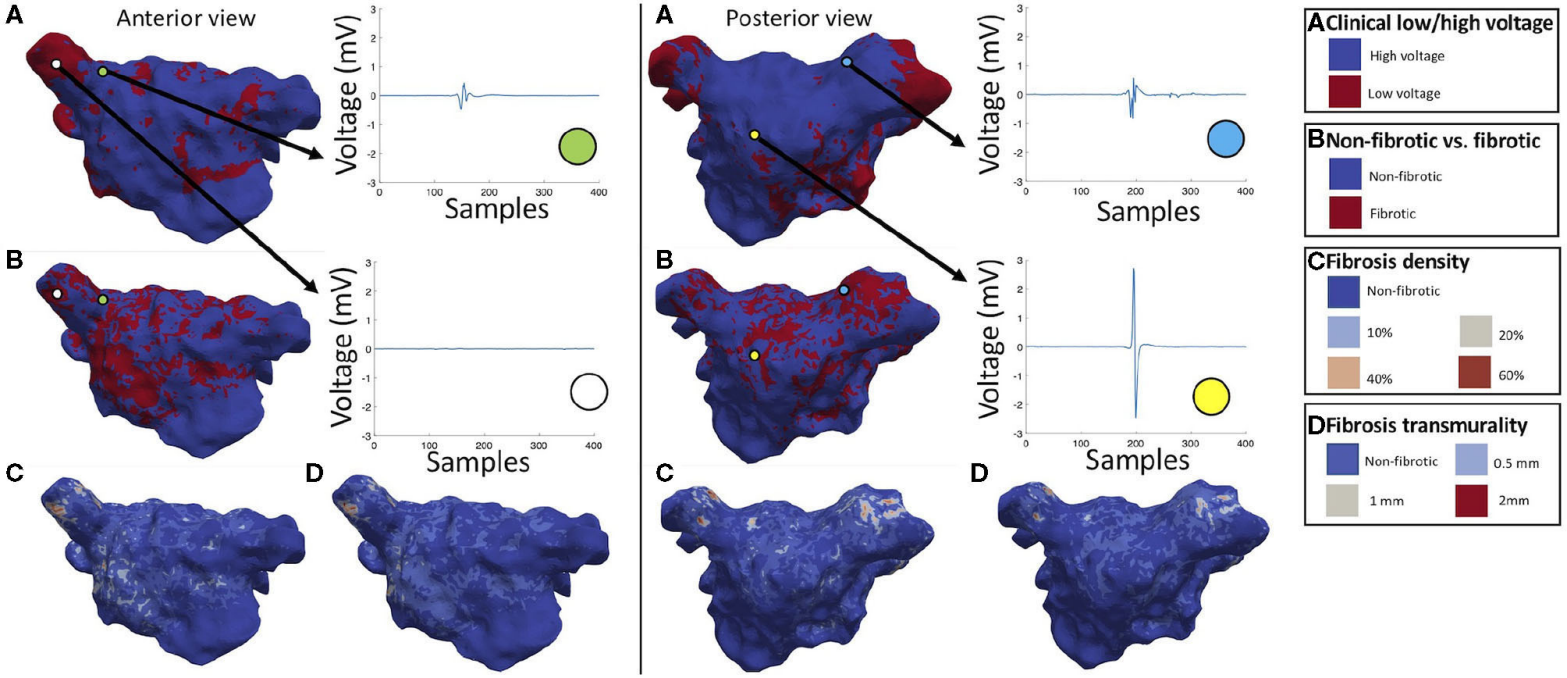
Anterior and posterior view of patient maps for clinical low/high voltage **(A)** and classification results for non-fibrotic vs. fibrotic **(B)**, fibrosis density **(C)**, and fibrosis transmurality **(D)**. The green dot represents a signal at the base of the pulmonary vein which was marked as high voltage and classified as subendocardial (0.5 mm) low density (10%) fibrotic tissue. The white dot refers to a signal recorded in the pulmonary vein classified as low voltage and high density (60%), transmural (2 mm) fibrotic tissue. The yellow dot represents a high voltage area identified as non-fibrotic and the light blue dot indicates a signal collected in the pulmonary vein annotated as high voltage and classified as low density (20%), partially transmural (1 mm) fibrotic tissue. Reused from Sánchez et al. (2021).

We applied the trained classifier to intracardiac signals measured in five patients of the test set of our cohort, which were not used to train the classifier, to create maps of atrial substrate characteristics. Figure 7 presents representative results for patient 1. The yellow dot (Figure 7A, posterior view) shows a signal annotated as high voltage and identified as non-fibrotic tissue by the classifier. Low voltage and high voltage areas determined by the clinical system using a cut-off value of 0.5 mV are shown in Figure 7A. The low voltage areas showed a mean dice similarity coefficient of 69.84 ± 0.03% with the predicted fibrotic areas for the five patients. Patients 1, 3, and 4 showed fibrotic areas mostly within the low voltage areas. Maps for the all the five patients are shown in Supplementary Figures 1–5. Figure 7B shows the classified fibrotic areas based on the signal features by the machine learning approach, where electrogram signals were fractionated and exhibited a longer activity duration independent from their peak-to-peak amplitude (Figure 7A, anterior view, green and white dot). Regions annotated as high voltage areas partly exhibited fractionated electrograms with a peak-to-peak voltage (1.4 mV) above the cut-off value of 0.5 mV (Figure 7A, posterior view, light blue dot) where these areas were classified as low density (20%) and partially transmural (1 mm) fibrosis. Fibrotic volume fraction was estimated using patient electrograms as input for the classifier (Figure 7C). In general for this patient cohort, high density was located at the core of fibrotic areas. Furthermore, Figure 7D shows the classification of different transmuralities. Fully transmural fibrosis was predominantly found in areas of high fibrotic density. Thus, not all high-density fibrotic areas are entirely transmural. In contrast to patient 3, patient 5 had a low similarity (58.76%) of low voltage and fibrotic areas due to a generally low peak-to-peak voltage in the electrograms (Supplementary Figure 5).

## 4. Discussion

We investigated the effect of fibrosis on intracardiac electrogram signals using computational models and trained machine learning algorithms using a combined *in vivo* and *in silico* dataset to classify the tissue according to fibrosis density and transmurality. We found that (i) detailed bidomain models in combination with models of clinical noise can reproduce clinical electrograms with high fidelity; (ii) complexity measurements help characterize fibrotic tissue from electrograms. Sample entropy and spectral entropy were the most distinguishing features to characterize fibrosis density, while fibrosis transmurality was identified by sample entropy and peak-to-peak amplitude; (iii) machine learning classifiers can characterize and distinguish tissue properties and quantify the amount of fibrosis density and transmurality from intracardiac signals with high accuracy and overcome the use of a single voltage cut-off value to localize arrhythmogenic substrate.

Bidomain simulations can reproduce the biophysical phenomenon of cardiac depolarization and the effect of mapping catheters on the electrograms. Bishop et al. demonstrated that including an extracellular medium induces the bath-loading effect, which impacts conduction velocity and translates to changes of electrogram morphology (Bishop and Plank, 2011). Our results show the effect of cylindrical metal electrodes on simulated signals. The high electrode conductivity markedly influences the electrogram slope as it acts as a current sink for the tissue underneath. Additionally, by using realistic geometries of catheters, spatial resolution is taken into account by preserving a realistic spacing of catheter electrodes. Moreover, the impact of the directionality of the propagating wave on bipolar electrograms was taken into account by stimulation from three different sites as previously discussed by Hwang et al. (2019).

Several studies investigated the influence of noise on simulated electrograms (Sameni et al., 2007; Frisch et al., 2020). Our simulated signals were able to reproduce the recorded clinical signals more realistically compared to simulations that do not consider the effect of the catheter and clinical noise. Simulated signals, even with noise, had a higher (Figure 4C) amplitude than clinically measured signals in line with previous reports by Keller et al. (2014). These higher amplitudes are likely due to two factors: Firstly, the catheter was placed directly on the surface of the tissue with perfect contact. Secondly, intracellular conductivity, which is related to the tissue's conduction velocity, can considerably increase the amplitude of the simulated signal. For this reason, our study included two different conduction velocities in the range of previously reported values for patients with persistent AF (McDowell et al., 2013).

Complexity measurements obtained from simulated intracardiac signals help understand the electrophysiology and the fibrotic tissue structural characteristics. Other studies showed that Shannon entropy and fractal dimensions help to localize the core of rotational activity (Cirugeda-Roldán et al., 2015; Rottmann et al., 2015). Cirugeda-Roldán et al. (2015) showed that sample entropy is a robust feature to classify complex fractionated electrograms. Our findings show that sample entropy, as well as spectral entropy, are robust morphological features to characterize fibrotic substrate and are less influenced by noise compared to the other entropy measures calculated in this study.

Our results show how *in silico* experiments can be used to generate realistic data for measurements that are difficult to obtain *in vivo*. Computational cardiac modeling can considerably accelerate the process of designing and evaluating medical devices, including mapping systems and software to treat patients with cardiac arrhythmia. The American Society of Mechanical Engineers (ASME) Verification and Validation Subcommittee standard V&V40 (Verification and Validation in Computational Modeling of Medical Devices) outlines credibility requirements of a computational model based on risk. We started by defining two questions of interest (“Can synthetic data be used to train a classifier to locate fibrotic tissue and quantify its characteristics?” and “Can a hybrid dataset approach predict the electrical characteristics to support ablation therapy?”). These guiding questions helped define the required model level of detail for the *in silico* experiment. In the next step, we established the risk-informed credibility of using a full bidomain model to simulate electrograms and using them to generate a hybrid dataset that combines clinical and synthetic signals. Risk-informed assessment defined the level of uncertainty and the model's complexity based on the context of use (CoU) of the *in silico* experiments.

In this pilot study, the CoU of the model is to generate a hybrid dataset to train a classifier to locate and quantify fibrotic tissue in clinical data. Different fibrosis modeling strategies change the dynamics of the electrical propagation as described by Roney et al. (2018), which influences the electrogram morphology. Fibrosis modeling uncertainty was reduced by considering several realizations of random uniformly distributed collagen fibers with different volume fraction and transmurality. We overcome the limitation of catheter geometry and wavefront direction by including two models of commercially available catheters and pacing from three different locations (Hwang et al., 2019). Two different human atrial cardiomyocyte models were considered to minimize the uncertainty of the action potential morphology influence on the electrogram. Moreover, an autoregression model of clinically measured noise artifacts was created. The modeled clinical noise in combination with the simulated electrograms reduced the uncertainty of simulated with respect to measured electrograms. Considering all the above mentioned points, the risk-informed assessment of using *in silico* experiments to characterize the fibrotic substrate was defined as medium.

Driven by the risk-informed assessment, we established the credibility of our modeling methodology. Model credibility refers to the trust in the predictive capability of a model for a specific CoU. openCARP source code and calculations are verified as described by Niederer et al. (2011). The model was validated using the clinical electrograms for high voltage areas. In combination with the noise model, the bidomain model reproduced the clinical signals with a mean correlation of 91.13 ± 0.05%. The strong correlation between *in silico* electrograms and *in vivo* measurements increased the confidence in the model.

With the increasing number of data available, data-driven approaches can help to improve patient's diagnosis and therapies. Several studies used data-driven approaches with clinical data to characterize electrocardiogram signals measured on the body surface (Yaghouby et al., 2010; Rodrigues et al., 2017; Zhang et al., 2018; Petmezas et al., 2021). Sahli Costabal et al. (2018) used a hybrid dataset approach to interpret activation times during AF and Lozoya et al. (2019) showed how model-based feature augmentation can help to plan the targets for ablation therapy. We developed a detailed *in silico* setup as a perfectly controlled testing environment to understand intracardiac signals recorded with two different commercial catheters. Furthermore, we trained a decision tree classifier using clinical and simulated data to characterize signals based on complexity measurements. Decision trees offer a comprehensible structure to follow the decisions taken for the classification. All three classifiers had high accuracy, despite overlapping features for different degrees of transmurality (Figure 5B), the combined features used to train all decision tree classifiers distinguished non-fibrotic tissue, fibrosis volume fraction, and all three different transmuralities of fibrosis from electrogram signals. Our results suggest that combining clinical and simulated data helps to characterize electrical tissue properties more accurately than using synthetic data alone. In future work, the classifier could be extended to include more training signals recorded directly at the surface of the tissue and at certain distances above the tissue to increase the performance when there is non-contact of the catheter with the tissue surface.

Different ablation strategies target fibrotic areas by ablating or isolating them (Hinderer and Schenke-layland, 2019). Both techniques rely on a voltage cut-off value for the identification of possible fibrotic areas. While ablating fibrotic areas try to homogenize the fibrotic substrate, isolation encloses the fibrotic regions and connects them to the pulmonary vein isolation lines to prevent a potential proarrhythmic effect. This suggests that identifying fibrotic tissue through electroanatomic mapping is essential, and the choice of a single voltage cut-off value may not be sufficient to decrease the recurrence of arrhythmia (Jadidi et al., 2016). Gutbrod et al. (2015) showed the importance of fibrosis transmurality for electric propagation during AF. Using a hybrid dataset approach, our findings can help to standardize the identification of non-fibrotic vs. fibrotic areas and provide valuable information on the fibrotic tissue characteristics such as fibrosis density and transmurality. Several studies have shown that low-density fibrosis can modify the propagation and initiate or maintain arrhythmia (Kazbanov et al., 2016; Jadidi et al., 2020). High-density fibrotic areas are prone to be a point of anchor for rotational activity (Alonso and Bär, 2013; Krul et al., 2015; Deng et al., 2017; Roy et al., 2018) while low-density fibrosis micro-structure can alter the propagation pattern and maintain reentry (Balaban et al., 2018; Campos et al., 2019). The trained classifier was used on five patients from the test set of our patient cohort to distinguish and characterize fibrotic tissue. For clinical data, not all low voltage areas were marked as fibrosis when using a single cut-off value. Areas with low-density (10%) subendocardial fibrosis (0.5 mm) were annotated as high voltage areas when using a single peak-to-peak cut-off value of 0.5 mV. Therefore, the use of hybrid datasets and data-driven approaches could help to estimate the fibrotic tissue characteristics to support the planning of ablation therapy. The medium-range dice coefficient (0.7) indicates that low voltage areas are one of the main indicators for fibrotic tissue but synergistic combination of multiple features in e.g., a decision tree classifier, can give a more comprehensive view beyond purely voltage-based tissue characterization.

Our results show that current clinical standards for substrate mapping using bipolar voltage alone are not sufficient to characterize the atrial fibrillation substrate comprehensively. Machine learning algorithms trained using hybrid datasets and multi-features obtained from intracardiac signals may overcome these limitations providing fibrosis density and transmurality maps. This may lead to optimized therapeutic approaches.

Our modeling approach does not capture the influence of the atrial anatomy and the tissue thickness heterogeneity. Nevertheless, our hybrid dataset approach tries to minimize this effect by including clinical signals from different patient. Furthermore, increasing the catheter to tissue distance decreases the accuracy of the classifier. The effect of the distance can likely be minimized if the dataset is extended to also include signals that were acquired at a certain distance to the cardiac tissue. Additionally, we only consider a homogeneous distribution of fibers from the endocardium to the epicardium, which may not represent the heterogeneous tissue architecture observed in some regions of the atria. The fibrotic regions were homogeneous and all electrodes were located inside the fibrotic area. We did not consider the effect of electrodes located at the border, which could result in more complex bipolar signals. We did not include any effect of inflammation-induced paracrine remodeling or myofibroblast interaction (Sánchez et al., 2019a). While our approach shows promising results and highlights the essential features of intracardiac signals to characterize atrial substrate, validation through independent experimental and clinical data is desirable. Future studies could include LGE-MRI data to validate the proposed approach and explore the arrangement of the fibrotic tissue effect on the electrogram morphology (Sánchez et al., 2019b).

Our modeling approach successfully answered the question of interest: A classifier can be trained using clinical and simulated data to characterize the cardiac substrate to support ablation therapy by providing fibrosis density and transmurality maps. Moreover, the credibility assessment showed that detailed cardiac modeling could be a valuable framework. In the future, classifiers to predict cardiac tissue characteristics could be integrated into clinical electroanatomic mapping systems. Finally, our study emphasizes the potential of *in silico* experimentation and data-driven approaches for characterizing the patient's substrate and demonstrates the potential of software tools to support medical decisions during the procedure.

## Data Availability Statement

The datasets presented in this study can be found in online repositories. The names of the repository/repositories and accession number(s) can be found at: https://github.com/jorge221/fibrosis_estimation.

## Ethics Statement

The studies involving human participants were reviewed and approved by Institutional Review Board of Freiburg University. The patients/participants provided their written informed consent to participate in this study.

## Author Contributions

JSá, JSa, BT, OD, and ALo designed the study. JSá conducted the simulations and analyzed the simulation results. GL and JSá designed the machine learning algorithm. MN, LU, and JSá processed the clinical signals and generated the clinical noise model. ALu performed the catheter mapping procedures and contributed to interpretation of the results. All authors reviewed the manuscript.

## Conflict of Interest

The authors declare that the research was conducted in the absence of any commercial or financial relationships that could be construed as a potential conflict of interest. The handling editor declared a past collaboration with the authors, BT and JSa.
