# Supplementary material for: Using Machine Learning to Characterize Atrial Fibrotic Substrate From Intracardiac Signals With a Hybrid in silico and in vivo Dataset
Source: Front Physiol. 2021 Jul 5;12:699291. doi: 10.3389/fphys.2021.699291 (PMC8287829; doi:10.3389/fphys.2021.699291)
Supplement: Supplementary file 1 [file Data_Sheet_1.PDF]

Supplementary Material

1 SUPPLEMENTARY FIGURES

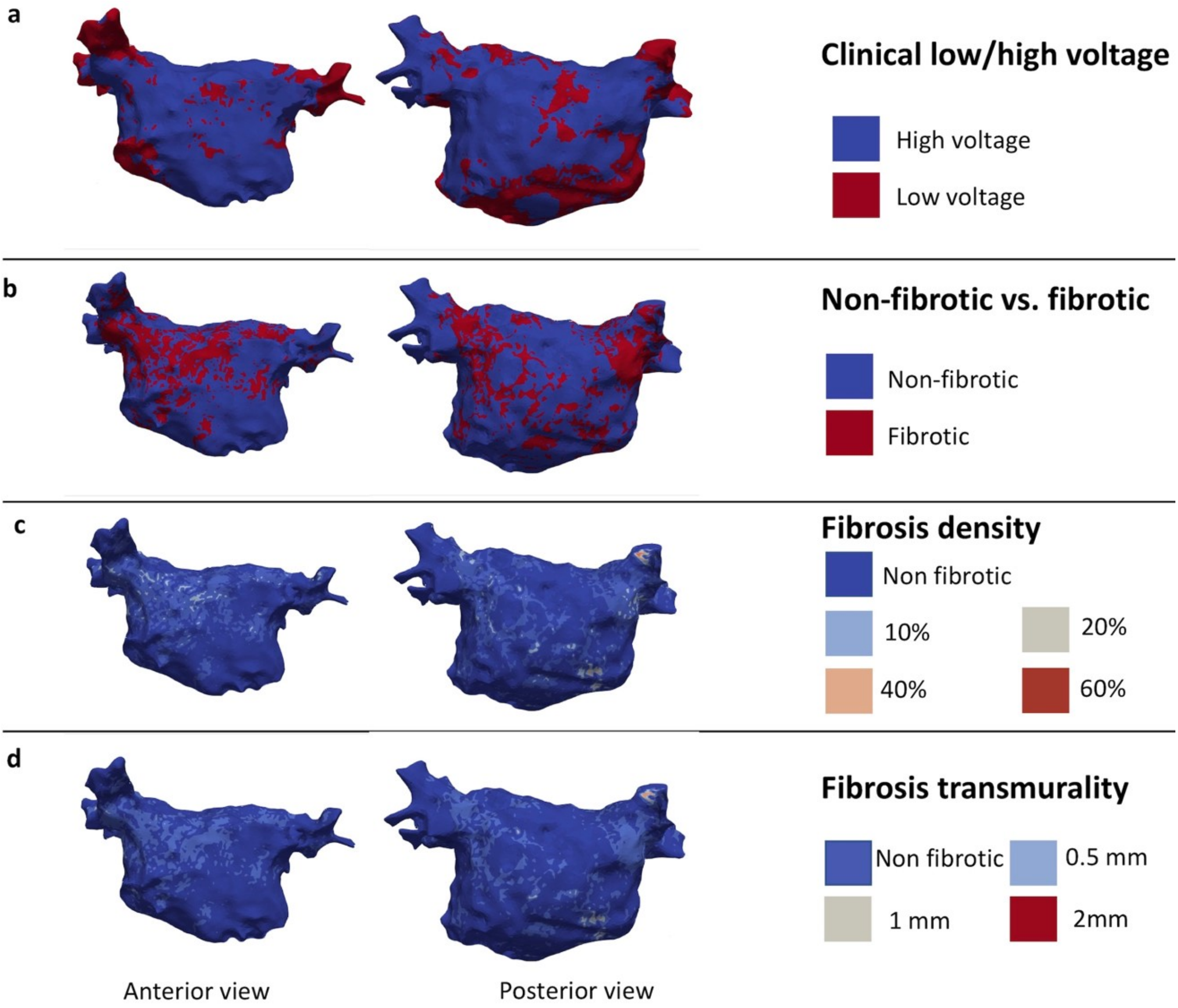

Figure S1. Anterior and posterior view of patient 1 maps for clinical low/high voltage (a) and classification results for non-fibrotic vs. fibrotic (b), fibrosis density (c), and fibrosis transmurality (d).

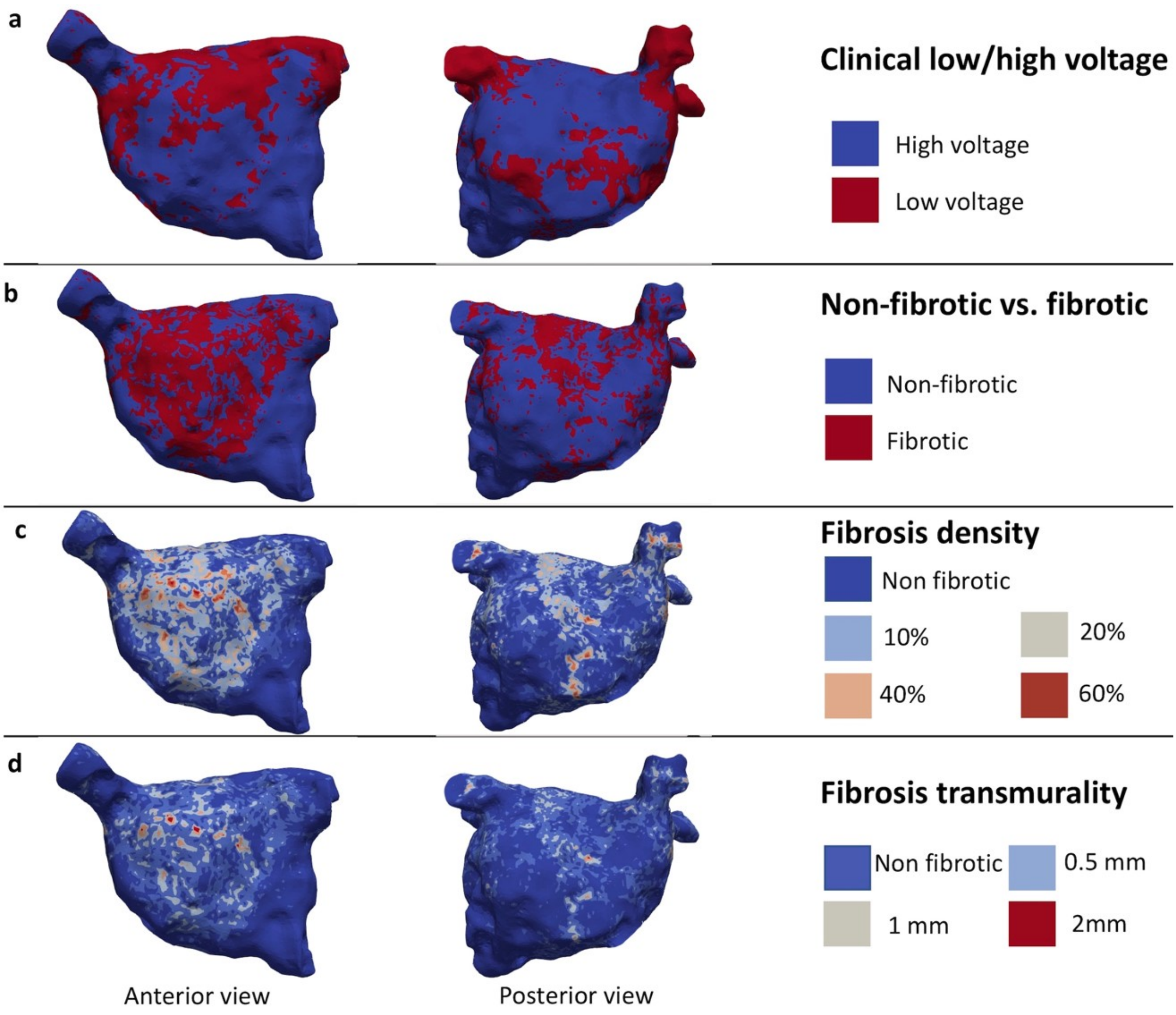

Figure S2. Anterior and posterior view of patient 2 maps for clinical low/high voltage (a) and classification results for non-fibrotic vs. fibrotic (b), fibrosis density (c), and fibrosis transmurality (d).

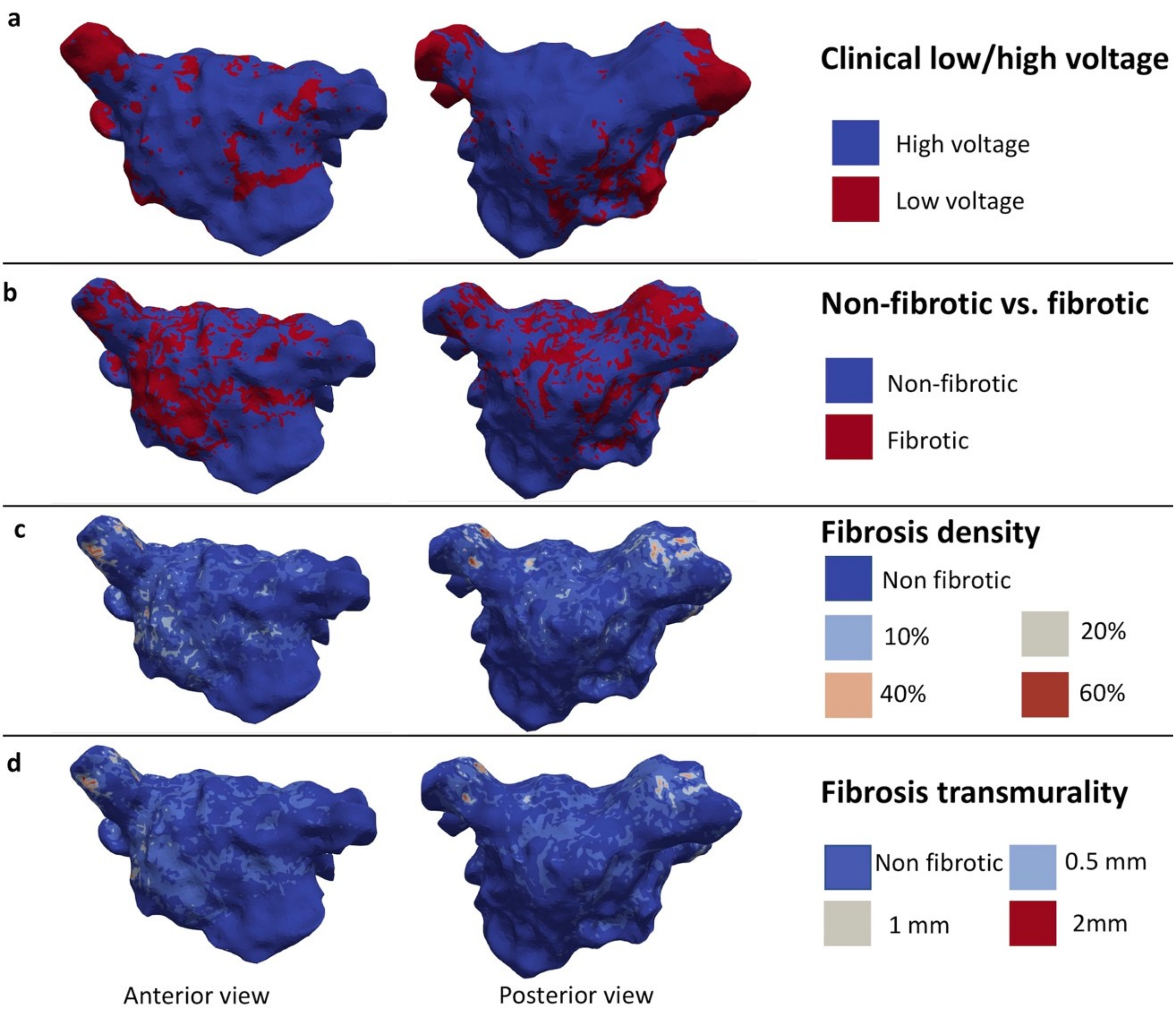

Figure S3. Anterior and posterior view of patient 3 maps for clinical low/high voltage (a) and classification results for non-fibrotic vs. fibrotic (b), fibrosis density (c), and fibrosis transmurality (d).

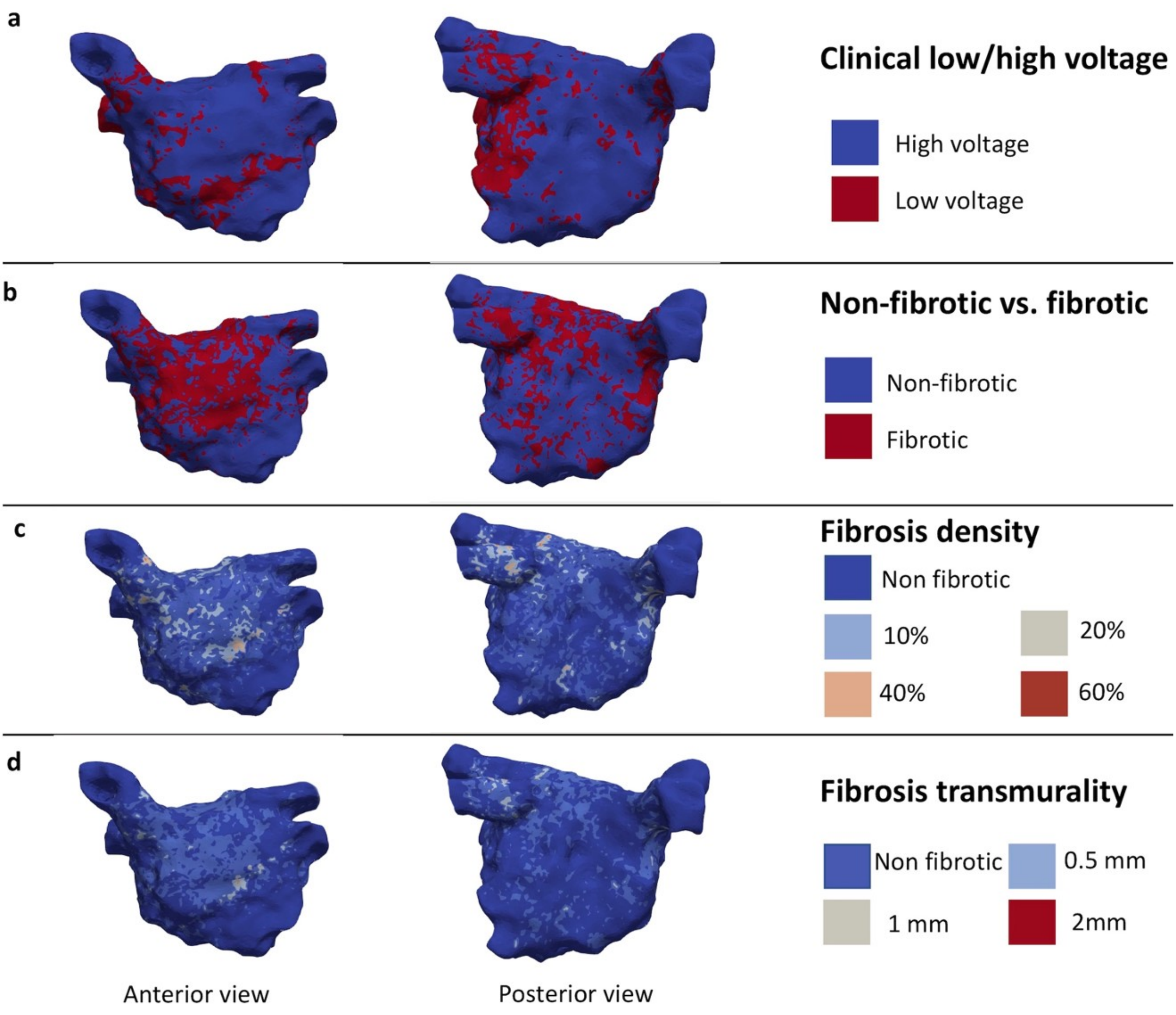

Figure S4. Anterior and posterior view of patient 4 maps for clinical low/high voltage (a) and classification results for non-fibrotic vs. fibrotic (b), fibrosis density (c), and fibrosis transmurality (d).

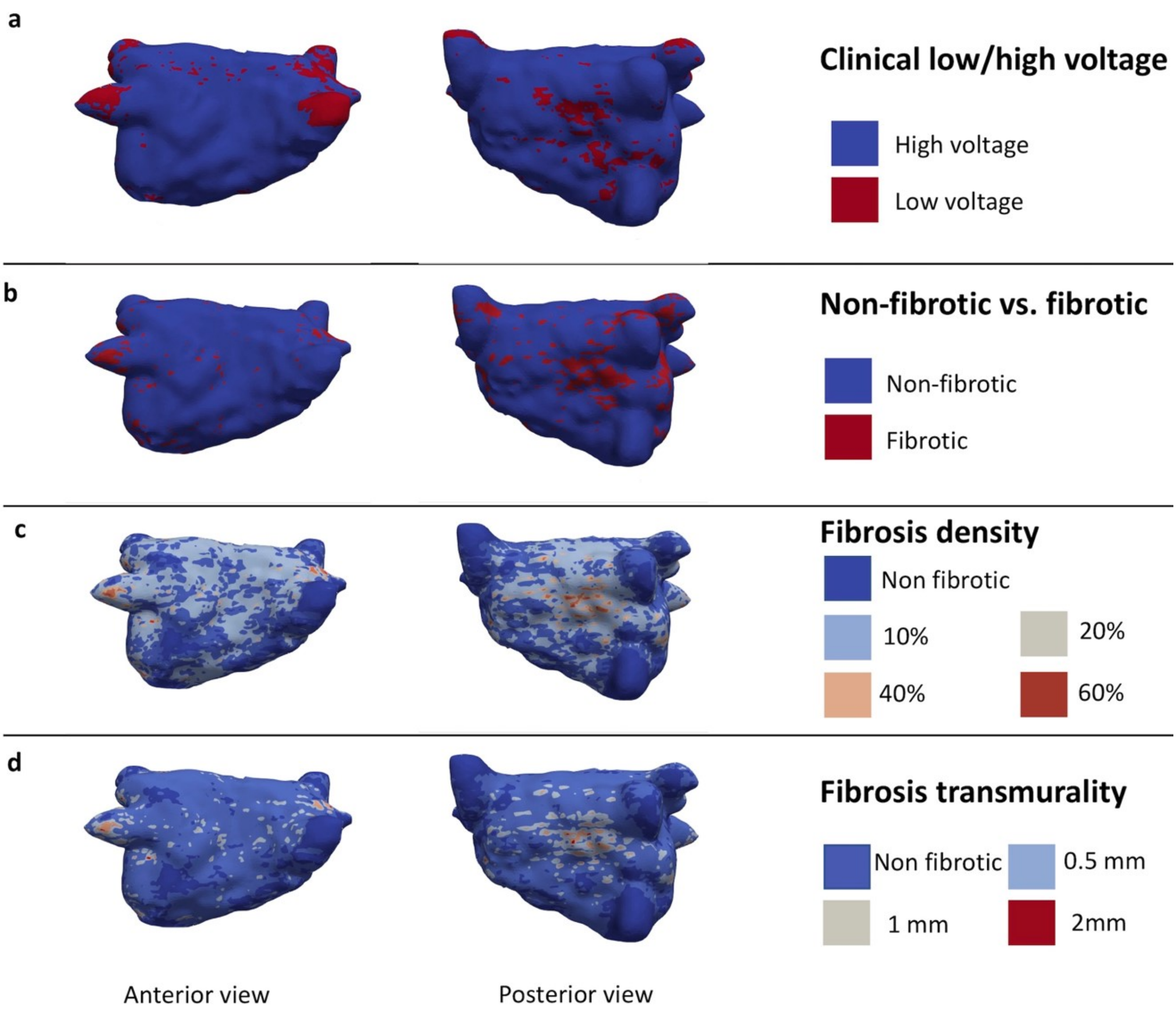

Figure S5. Anterior and posterior view of patient 5 maps for clinical low/high voltage (a) and classification results for non-fibrotic vs. fibrotic (b), fibrosis density (c), and fibrosis transmurality (d).
